# NZ28-induced inhibition of HSF1, SP1 and NF-κB triggers the loss of the natural killer cell-activating ligands MICA/B on human tumor cells

**DOI:** 10.1007/s00262-015-1665-9

**Published:** 2015-02-18

**Authors:** Daniela Schilling, Annett Kühnel, Fabian Tetzlaff, Sarah Konrad, Gabriele Multhoff

**Affiliations:** 1 Department of Radiation Oncology, Klinikum rechts der Isar, Technische Universität München, TUM, Ismaningerstr. 22, 81675 Munich, Germany; 2 Helmholtz Center Munich, German Research Center for Environmental Health – Institute of Biological and Medical Imaging, Munich, Germany

**Keywords:** Heat shock factor 1 (HSF1), MICA/B, Natural killer (NK) cells, NKG2D, NVP-AUY922, NZ28

## Abstract

The activity of natural killer (NK) cells is regulated by activating and inhibiting receptors, whereby the C-type lectin natural killer group 2D (NKG2D) receptor serves as the major activating receptor on NK cells which recognizes major histocompatibility class I chain-related proteins A and B (MICA/B). The MICA/B expression has been described to be regulated by the transcription factor heat shock factor 1 (HSF1). Inhibition of heat shock protein 90 (Hsp90) is known to induce the heat shock response via activation of HSF1 which is associated with tumor development, metastasis and therapy resistance and also with an increased susceptibility to NK cell-mediated lysis. Therefore, we compared the effects of Hsp90 inhibitor NVP-AUY922, HSF1 inhibitor NZ28 and HSF1 knockdown on the sensitivity of lung (H1339) and breast (MDA-MB-231, T47D) cancer cells to NK cell-mediated cytotoxicity and the expression of the NKG2D ligands MICA/B. Although NVP-AUY922 activates HSF1, neither the MICA/B surface density on tumor cells nor their susceptibility to NK cell-mediated lysis was affected. A single knockdown of HSF1 by shRNA decreased the surface expression of MICB but not that of MICA, and thereby, the NK cell-mediated lysis was only partially blocked. In contrast, NZ28 completely blocked the MICA/B membrane expression on tumor cells and thereby strongly inhibited the NK cell-mediated cytotoxicity. This effect might be explained by a simultaneous inhibition of the transcription factors HSF1, Sp1 and NF-κB by NZ28. These findings suggest that new anticancer therapeutics should be investigated with respect to their effects on the innate immune system.

**Electronic supplementary material:**

The online version of this article (doi:10.1007/s00262-015-1665-9) contains supplementary material, which is available to authorized users.

## Introduction

Inhibition of heat shock protein 90 (Hsp90) which promotes tumor cell survival and proliferation by stabilizing multiple oncogenic client proteins is a promising concept to overcome resistance of tumor cells to anticancer therapies. Numerous inhibitors of Hsp90 are currently tested in clinical trials. A negative side effect of Hsp90 inhibition is the activation of heat shock factor 1 (HSF1) which induces the expression of other cytoprotective stress proteins including Hsp70. Consequently, inhibition of Hsp70 or HSF1 has been shown to improve the anticancer effectivity of Hsp90 inhibitors [1–5]. With respect to ionizing irradiation, a simultaneous treatment of tumor cells with the HSF1 inhibitor NZ28 [5] and the Hsp90 inhibitor NVP-AUY922 has been shown to potentiate the radiosensitizing effect mediated by NVP-AUY922 alone (unpublished data). Herein, we study how modulators of the heat shock response can affect natural killer (NK) cell-mediated immunity. The cytolytic activity of NK cells is regulated by a fine balance of activating and inhibitory NK cell receptors. Among others, the C-type lectin natural killer group 2D receptor (NKG2D) acts as an activating receptor which recognizes the major histocompatibility class I chain-related proteins A and B (MICA/B). An enhanced sensitivity of tumor cells to NK cell-mediated lysis has been attributed to an increased membrane expression density of the NKG2D ligands MICA and MICB [6–9]. Vice versa, a reduced MICA/B membrane expression on tumor cells impairs the recognition by NK cells and promotes tumor immune escape [10]. The molecular mechanisms that regulate the expression of MICA/B on tumor cells are not completely understood. In the promoter regions of MICA and MICB, heat shock elements (HSE) which are similar to those of the *hsp70* genes have been found [11]. Stress such as heat shock induces the binding of the transcription factor HSF1 to the HSE in the promoter region of MICA/B and thus up-regulates mRNA and protein expression of MICA/B [12, 13]. Inhibitors of Hsp90 which are also known to activate HSF1 increase the expression of MICA/B in a variety of multiple myeloma cells [6].

However, besides HSF1, other factors such as the transcription factor SP1 which binds constitutively to the MICA/B promoter [12] have been described to participate in the transcriptional regulation of MICA/B. Histone deacetylase inhibition (HDAC) can increase the binding of HSF1 and SP1 to the promoter of MICA/B and thus results in an increased membrane MICA/B expression [8, 14]. In endothelial cells, a treatment with TNF-α induces binding of the transcription factor NF-κB to the MICA promoter and thereby causes an up-regulated expression of MICA [15].

In the present study, we were interested to analyze the effects of HSF1 activation (Hsp90 inhibitor NVP-AUY922) and inhibition (NZ28, HSF1 knockdown) in different human cancer cells on the NK cell ligands MICA/B and its consequences on NK cell-mediated lysis. Our data demonstrate that Hsp90 inhibition alters neither the MICA/B surface density nor the sensitivity of the tumor cells to NK cell-mediated lysis. A knockdown of HSF1 decreases the membrane expression of MICB but not that of MICA, whereas a treatment with NZ28 inhibits the expression of both, MICA and MICB on the surface of the investigated tumor cells. In line with these findings, the loss of MICA and MICB on NZ28-treated tumor cells resulted in a complete inhibition of the NK cell-mediated cytotoxicity, whereas down-regulation of MICB by HSF1 knockdown resulted in a partial reduction in lysis mediated by NK cells. We also could show that NZ28 inhibits not only HSF1 but also other transcription factors such as NF-κB and Sp1 which are responsible for the expression of MICA/B.

## Materials and methods

### Reagents

10 mM stock solutions of NZ28 (J. Yaglom and M. Sherman; Boston University School of Medicine, USA) and NVP-AUY922 (Novartis) were prepared in 100 % DMSO. Dilutions were performed in PBS. A vehicle control with the respective amount of DMSO diluted in PBS was tested in all experiments to exclude an effect of DMSO itself (maximal 0.2 %).

### Cells and cell culture

The human lung (H1339) and breast (MDA-MB-231, T47D) cancer cell lines were cultured as described previously [16, 17]. Cells were routinely checked for mycoplasma contamination. The authenticity of the cell lines was tested by the DSMZ (German collection of microorganisms and cell cultures).

### Retroviral vectors and infection

For knockdown of HSF1, RNAi-Ready pSIREN-RetroQ vector with puromycin resistance (BD Biosciences) was used. Target sequence for HSF1 small interfering RNA was 5′-TATGGACTCCAACCTGGATAA-3′ [5]. Retroviruses were produced by transfection of Phoenix cells with pSIREN-RetroQ/HSF1 shRNA (shHSF1) or pSIREN-RetroQ (control) (provided by J. Yaglom and M. Sherman, Boston University School of Medicine, USA) using Ca phosphate. Tumor cells were infected with virus containing supernatants in the presence of 10 µg/ml polybrene. Selection was performed with 2 µg/ml puromycin.

### Western blot analysis and ELISA

Cells were lysed in TBST buffer as described previously [18]. The protein content in the cell lysates was determined using the BCA™ Protein Assay Kit (Pierce). On immunoblots, proteins were detected with antibodies against HSF1 (ADI-SPA-901; Enzo Life Sciences), HSF1 phospho S326 (pHSF1) (ab76076; abcam), Hsp70 (ADI-SPA-810; Enzo Life Sciences) and β-actin (A5316; Sigma-Aldrich).

MICA and MICB concentrations in the cell lysates were measured by ELISA (R&D Systems), and the concentrations were calculated relative to the total protein content of each sample.

### Luciferase assay

Cells were transfected with an inducible transcription factor-responsive (HSF1, Sp1, NF-κB) firefly luciferase construct (Qiagen). The luciferase activity was measured using the Dual-Glo Luciferase assay system (Promega). A constitutive Renilla luciferase construct served as an internal control for normalizing transfection efficiencies, cell viability and cell numbers.

### Flow cytometry

Cells were incubated with the APC-conjugated MICA (clone 159227, FAB1300A, R&D Systems) and MICB antibodies (clone 236511, FAB1599A, R&D Systems) or the corresponding isotype-matched control antibody (clone 133303, IC0041A, R&D Systems) for 30 min at 4 °C. Dead cells were stained with propidium iodide, and only viable cells were analyzed on a FACSCalibur flow cytometer (BD Biosciences).

### NK cell isolation

NK cells were generated by a CD3/CD19 depletion of PBMCs from healthy human volunteers using a magnetic separation method (Miltenyi Biotec). The purity of NK cells (89.9 % of lymphocytes) was determined by flow cytometry with antibodies against CD19 (#555413, BD Biosciences), CD3 (#345766, BD Biosciences) and CD56 (#345811, BD Biosciences).

### NK cell cytotoxicity

NK cells were stimulated with 100 IU/ml IL-2 (Novartis) for 4 days. Activation of NK cells was checked by flow cytometry with antibodies against CD94 (#555888, BD Biosciences), NKG2D (FAB139P, R&D Systems) and CD56 (#345811, BD Biosciences). Their cytotoxic activity against differentially pre-treated tumor cells was determined either by europium or by CD107 degranulation assay. During the co-incubation of tumor and NK cells, no drugs were added.

For the europium assay, the tumor cells were labeled with BATDA (Perkin Elmer) and then co-incubated with NK cells at different ratios in a V-bottom 96-well plate in 200 µl-medium. After a 4-h co-incubation period at 37 °C, 25 µl of supernatants were transferred into ELISA plates containing 200 µl europium solution (Perkin Elmer). The time-resolved fluorescence was measured using a Victor X4 plate reader (Perkin Elmer).

In the degranulation assay, the cytotoxicity of NK cells was determined by measuring the cell surface expression of the lysosomal marker, CD107a, which correlates with NK cell cytotoxicity [19]. Tumor cells were mixed with NK cells at a ratio of 1:1 in a U-bottom 96-well tissue culture plate. Anti-CD107a-FITC (#555800, BD Biosciences) or the isotype-matched control antibody (#555748, BD Biosciences) was added. After 1 h, GolgiStop™ (BD Biosciences) was added and after a co-incubation period of 3 h, the cells were stained with anti-CD3-PerCP (#345766, BD Biosciences) and anti-CD56-APC (#555518, BD Biosciences) antibodies. The CD107a expression on the CD3^−^CD56^+^ NK cell population was determined by flow cytometry.

### Statistics

Statistical analysis was performed using SPSS 18.0.2 software (IBM). The unpaired Student’s *t* test was used to evaluate significant differences (*p ≤ 0.05, **p ≤ 0.01, ***p ≤ 0.001).

## Results

### NZ28 impairs susceptibility of tumor cells to NK cell-mediated lysis

**Fig. 1:**
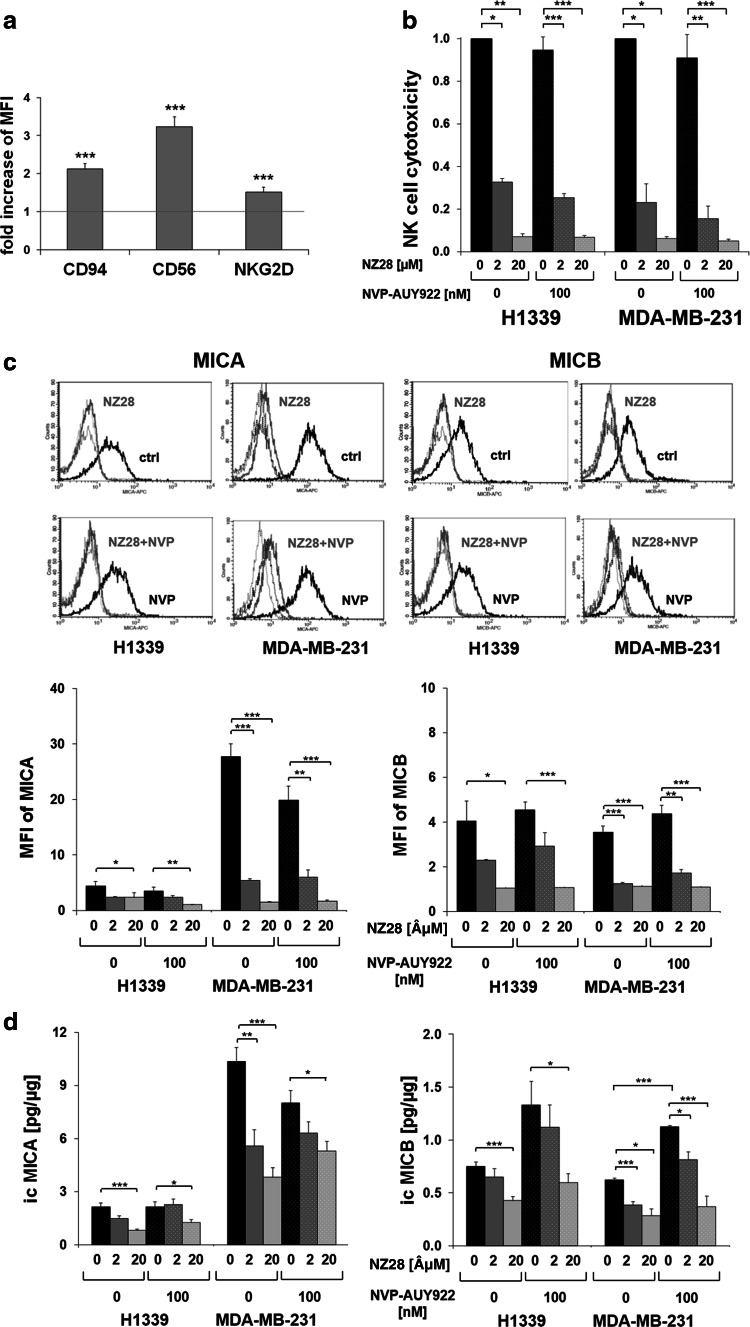
NZ28 impairs killing of tumor cells by activated NK cells. Tumor cells were treated with 2 or 20 µM NZ28 and/or 100 nM NVP-AUY922 for 24 h. DMSO (0.2 %)-treated cells served as control. NK cells were stimulated for 4 days with 100 U/ml IL-2. **a** The expression of CD94, CD56 and NKG2D on NK cells was determined by FACS analysis. The fold increase in mean fluorescence intensity (MFI) of cell surface markers on stimulated compared to unstimulated NK cells is shown. *Graphs* represent mean values of seven independent experiments ±SEM. *p ≤ 0.05, **p ≤ 0.01, ***p ≤ 0.001. **b** The cytotoxicity of activated NK cells was measured after a 4-h co-incubation with tumor cells by the CD107a degranulation assay. The relative cytotoxicity of activated NK cells incubated with differentially treated tumor cells compared to control cells is shown. *Graphs* represent mean values of 2–3 independent experiments ±SEM. *White dots* in the *bar graphs* indicate treatment of tumor cells with NVP-AUY922. *p ≤ 0.05, **p ≤ 0.01, ***p ≤ 0.001. **c** Tumor cells were stained with antibodies against MICA/B or the respective isotype control. Representative FACS analysis shows MICA (*left*) and MICB (*right*) surface staining on tumor cells. The *black solid lines* represent MICA or MICB staining of control cells (ctrl; *upper panels*) or cells treated with 100 nM NVP-AUY922 (NVP; *lower panels*). The *gray solid lines* represent MICA or MICB staining of cells treated with 20 µM NZ28 (NZ28; *upper panels*) or with 20 µM NZ28 plus 100 nM NVP-AUY922 (NZ28 + NVP; *lower panels*). *Dotted lines* represent the respective isotype controls. *Graphs* show mean values of at least two independent experiments ±SEM of the mean fluorescence intensity (MFI) of MICA and MICB. *White dots* in the *bar graphs* indicate treatment of tumor cells with NVP-AUY922. *p ≤ 0.05, **p ≤ 0.01, ***p ≤ 0.001. **d** The intracellular levels of MICA (icMICA, *left*) and MICB (icMICB, *right*) in tumor cells were determined by ELISA. The concentrations in the cell lysates were calculated relative to the total protein content of each sample. *Graphs* represent mean values of four independent experiments ±SEM. *White dots* in the *bar graphs* indicate treatment of tumor cells with NVP-AUY922. *p ≤ 0.05, **p ≤ 0.01, ***p ≤ 0.001

To investigate whether inhibitors of the stress response can modulate the sensitivity of human tumor cells to NK cell-mediated cytotoxicity, HSF1 inhibitor NZ28 and/or Hsp90 inhibitor NVP-AUY922-treated tumor cells were used as target cells for IL-2 (100 IU/ml)-activated NK cells. A successful stimulation of purified NK cells was shown by an up-regulated cell surface density of the NK cell markers CD94, CD56 and NKG2D (Fig. 1a) [20]. The cytotoxic activity of NK cells against H1339 (lung) and MDA-MB-231 (breast) cancer cells was determined by measuring the expression of the lysosomal marker CD107a [6, 19]. As shown in Fig. 1b, the NK cell-mediated cytotoxicity against H1339 and MDA-MB-231 tumor cells that were treated with 2 or 20 µM NZ28 was significantly reduced compared to untreated tumor cells (Fig. 1b). Treatment of tumor cells with 100 nM NVP-AUY922 alone did not affect the NK cell-mediated cytotoxicity, and a combined treatment with NZ28 and NVP-AUY922 did not reverse the immunosuppressing effect of NZ28. Similar findings were observed in another breast cancer cell line (T47D, supplementary Fig. 1a). A direct drug-induced cytotoxicity could be excluded since the drug concentrations which were used in the cytotoxicity assays did not affect cell viability of the tumor cells (supplementary Figs. 1b, 2).

### NZ28 suppresses the expression of activating NK cell ligands MICA and MICB

Hsp90 inhibitors such as NVP-AUY922 are known to activate HSF1, whereas NZ28 has been shown to inhibit the heat shock response by reducing the HSF1 activity [5, 21]. As HSF1 has been described to regulate the transcription of the NK cell ligands MICA and MICB [12, 13], we investigated whether treatment of tumor cells with NZ28 and/or NVP-AUY922 alters the cell surface density [mean fluorescence intensity (MFI)] of the NK cell ligands MICA and MICB on tumor cells.

To test whether detaching adherent tumor cells with trypsin is suitable to measure the MICA/B membrane expression, the surface expression of MICA/B on cells that were detached with trypsin and accutase was compared by FACS analysis. Accutase has been described to perform exceptionally well in detaching cells without altering the membrane expression of MICA/B [22]. As no differences were observed (supplementary Fig. 3), tumor cells treated for 24 h with NZ28 and/or NVP-AUY922 were detached with trypsin and the MICA/B membrane expression was determined by FACS analysis. Representative flow cytometric histograms are shown in Fig. 1c (upper part). Treatment of H1339 and MDA-MB-231 tumor cells with 100 nM NVP-AUY922 did not affect the MICA and MICB cell surface density (MFI) (Fig. 1c, lower part). In contrast, NZ28 significantly reduced the MICA and MICB membrane expression (MFI) in both tumor cell lines. In T47D tumor cells which lack a MICA membrane expression, the MICB expression was found to be strongly reduced upon treatment with NZ28 (supplementary Fig. 4).

In line with the membrane expression, the intracellular (ic) expression of MICA and MICB was significantly reduced in H1339 and MDA-MB-231 cells by NZ28 (Fig. 1d). The Hsp90 inhibitor NVP-AUY922 did not affect the icMICA levels but increased the icMICB levels.

**Fig. 2:**
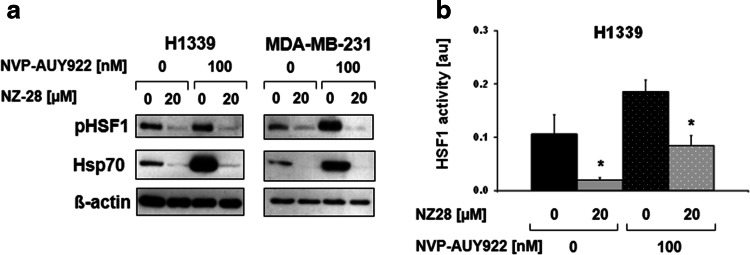
NZ28 reduces Hsp70 expression and HSF1 activity. **a** Representative HSF1 phospho S326 (pHSF1) and Hsp70 immunoblots of H1339 and MDA-MB-231 tumor cells treated with 20 µM NZ28 and/or 100 nM NVP-AUY922 for 24 h. DMSO (0.2 %) treated cells served as control. **b** Luciferase assay of H1339 cells transfected with a HSF1 responsive firefly luciferase construct and treated with 20 µM NZ28 and/or 100 nM NVP-AUY922 for 24 h. DMSO (0.2 %)-treated cells served as control. *White dots* in the *bar graphs* indicate treatment with NVP-AUY922. *Graphs* represent mean values ± SEM of at least three independent experiments. Significant differences are indicated (*p ≤ 0.05, **p ≤ 0.01)

The activation of HSF1 by NVP-AUY922 and the inhibition of HSF1 by NZ28 were proven by immunoblots and HSE-luciferase reporter assays. As expected, NVP-AUY922 increased, whereas NZ28 decreased phosphorylated HSF1 at serine residue 326 (pHSF1 Ser326) and Hsp70 expression as well as HSF1 activity (Fig. 2, supplementary Fig. 5).

### HSF1 depletion mimics NZ28 by impairing NK cell-mediated lysis

**Fig. 3:**
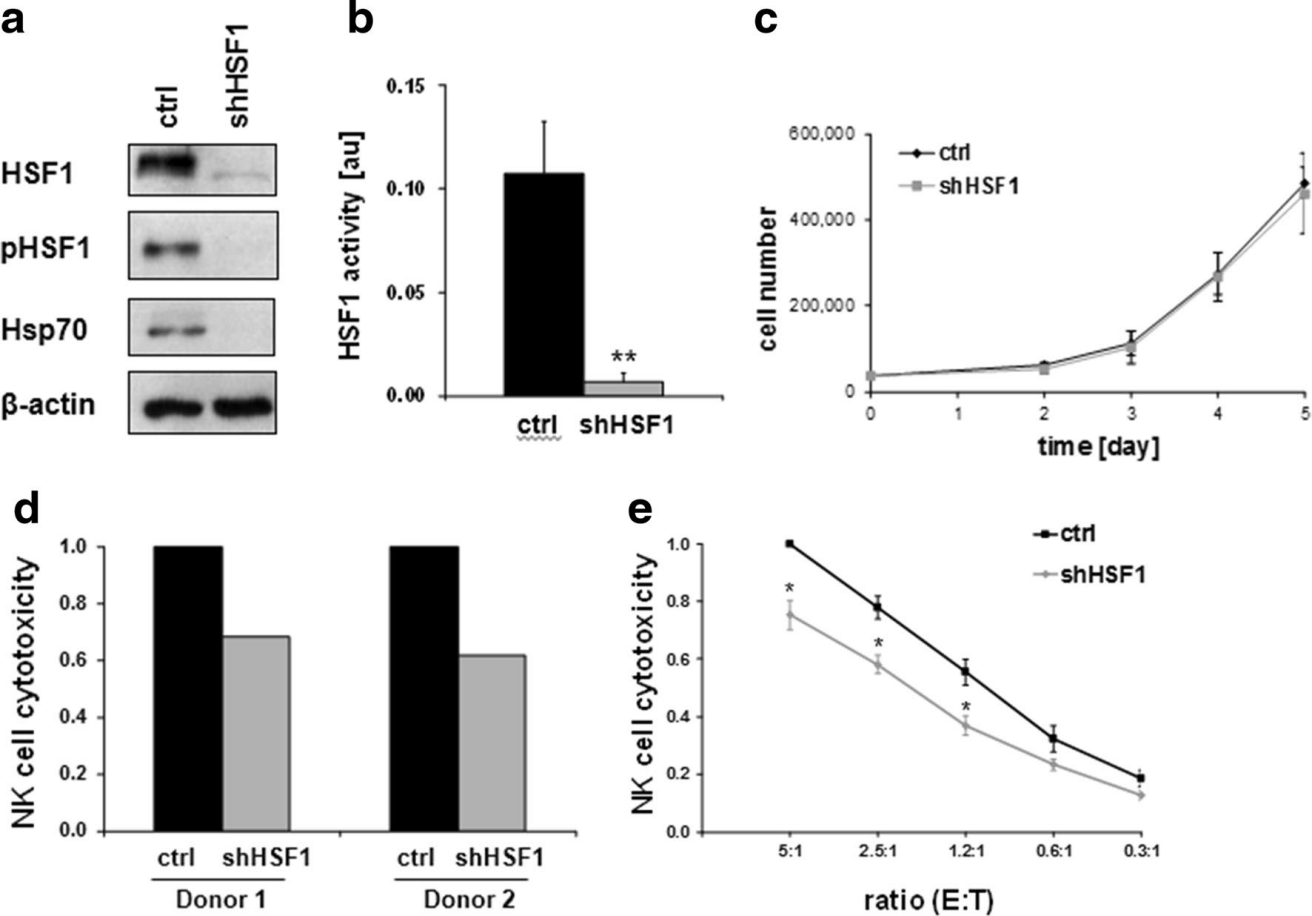
HSF1 depletion impairs lysis of H1339 tumor cells by activated NK cells. **a** Representative immunoblot showing the expression of HSF1, HSF1 phospho S326 (pHSF1), Hsp70 and ß-actin in H1339 cells transfected with control (ctrl) or HSF1 shRNA (shHSF1). **b** Luciferase assay of H1339 ctrl and shHSF1 cells transfected with a HSF1 responsive firefly luciferase construct. *Graphs* represent mean values ± SEM of five experiments. Significant differences are indicated (*p ≤ 0.05). **c** Growth curve of H1339 cells transfected with control (ctrl) or HSF1 shRNA (shHSF1). **d** NK cells from two different healthy donors were stimulated for 4 days with 100 U/ml IL-2. The cytotoxicity of IL-2-stimulated NK cells was measured after a 4-h co-incubation with H1339 tumor cells transfected with control (ctrl) or HSF1 shRNA (shHSF1) by the CD107a degranulation assay. The relative cytotoxicity of NK cells incubated with shHSF1 cells compared to NK cells incubated with control tumor cells is shown for each NK cell donor. **e** NK cells were stimulated for 4 days with 100 U/ml IL-2. The cytotoxicity of IL-2-stimulated NK cells was measured after a 4-h co-incubation with H1339 tumor cells transfected with control (ctrl) or HSF1 shRNA (shHSF1) by europium assay. The cytotoxicity of stimulated NK cells against control tumor cells at an E:T (effector to target) ratio of 5:1 was set to one. *Graphs* represent mean values ± SEM of at least three independent experiments. Significant differences between control and HSF1 knockdown cells are indicated (*p ≤ 0.05)

To investigate whether an inhibition of HSF1 can explain the effects of NZ28 on the NK cell-mediated cytotoxicity and MICA/B expression, we knocked down HSF1 in H1339 tumor cells by RNA interference. H1339 cells were infected either with a retrovirus encoding a specific shRNA against HSF1 (shHSF1) or with a control retrovirus (ctrl). The successful knockdown of HSF1 is shown in Fig. 3a. The HSF1 knockdown also strongly reduced the expression of Hsp70 (Fig. 3a) and of a HSE-luciferase reporter construct (Fig. 3b). Despite the HSF1 knockdown, tumor cell growth under standard cell culture conditions was not impaired (Fig. 3c).

To investigate whether depletion of HSF1 in H1339 tumor cells affects NK cell-mediated cytotoxicity, control and HSF1 knockdown tumor cells were used as target cells for IL-2-activated NK cells. The cytotoxicity of stimulated NK cells against shHSF1 knockdown tumor cells was strongly reduced compared to control cells as demonstrated by degranulation (Fig. 3d) and europium assay (Fig. 3e). However, the reduction in NK cell-mediated lysis was much more pronounced after treatment of the H1339 tumor cells with NZ28 (Fig. 1b) compared to that of a HSF1 knockdown (Fig. 3d).

### HSF1 regulates MICB but not MICA expression

**Fig. 4:**
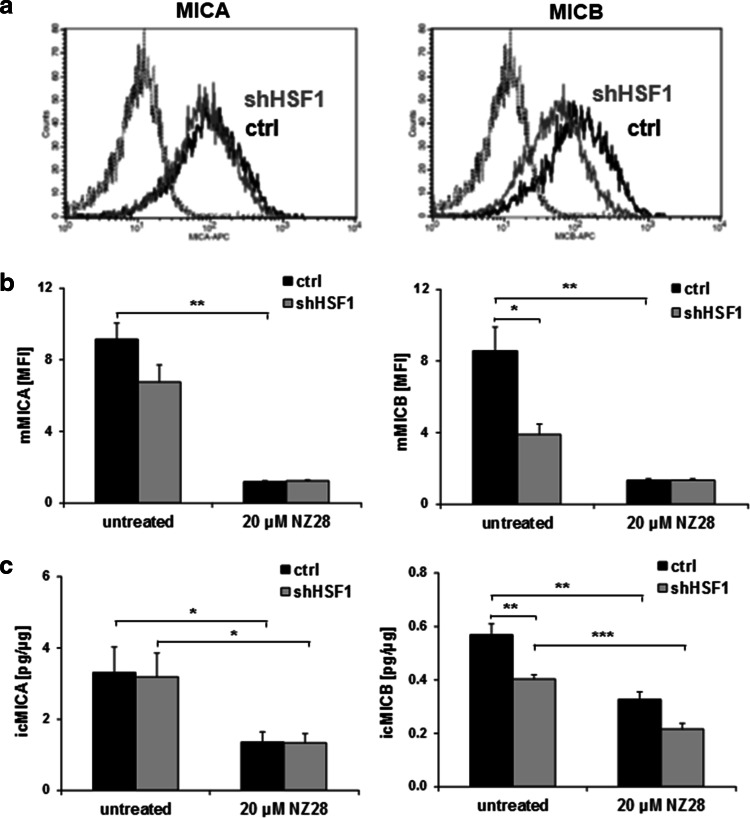
HSF1 knockdown reduces membrane and intracellular MICB levels. The expression of MICA and MICB on the membrane of H1339 tumor cells transfected with control (ctrl) or HSF1 shRNA (shHSF1) was determined by flow cytometry (**a**, **b**) and the intracellular expression by ELISA (**c**). **a** Representative FACS analysis showing MICA and MICB surface staining. The *black solid lines* represent MICA or MICB staining of control (ctrl) cells. The *gray solid lines* represent MICA or MICB staining of cells transfected with shRNA against HSF1 (shHSF1). *Dotted lines* represent the respective isotype controls. **b** The mean fluorescence intensity (MFI) of MICA and MICB on H1339 cells transfected with control (ctrl) or HSF1 shRNA (shHSF1) and treated with 20 µM NZ28 for 24 h is shown. *Graphs* represent mean values ± SEM of at least three independent experiments. Significant differences are indicated (*p ≤ 0.05, **p ≤ 0.01, ***p ≤ 0.001). **c** The intracellular levels of MICA (icMICA,* left*) and MICB (icMICB,* right*) of H1339 cells transfected with control (ctrl) or HSF1 shRNA (shHSF1) and treated with 20 µM NZ28 for 24 h were determined by ELISA. The concentrations in the cell lysates were calculated relative to the total protein content of each sample. *Graphs* represent mean values ± SEM of at least three independent experiments. Significant differences are indicated (*p ≤ 0.05, **p ≤ 0.01, ***p ≤ 0.001)

To ascertain whether the HSF1 knockdown has any impact on MICA/B levels, the membrane expression and ic expression of MICA/B were measured by FACS and ELISA, respectively. As shown in Fig. 4a, b, the HSF1 knockdown did not affect the MICA, but reduced the MICB surface density significantly. In line with the membrane expression, the ic MICB levels were significantly down-regulated by HSF1 knockdown, whereas the ic MICA expression remained unaffected (Fig. 4c). These data suggest that HSF1 regulates the transcription of MICB but not that of MICA.

### NZ28 inhibits activity of the MICA/B transcription factors NF-κB and Sp1

**Fig. 5:**
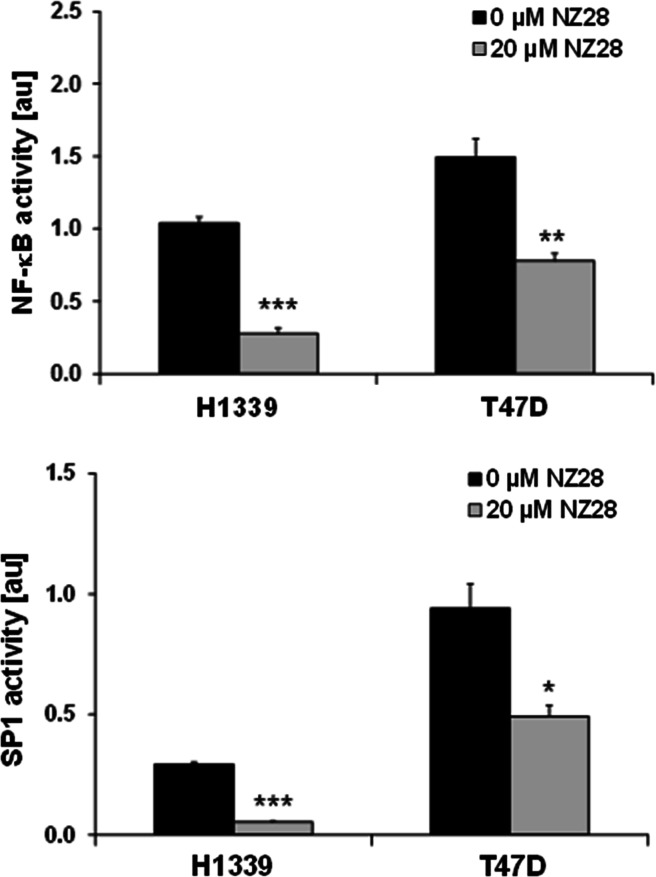
NZ28 reduces NF-κB and SP1 transcriptional activity in tumor cells. H1339 and T47D cells were transfected with reporter plasmids that contain NF-κB and SP1 responsive firefly luciferase constructs and treated with 20 µM NZ28 for 24 h. DMSO (0.2 %)-treated cells served as control (0 µM NZ28). Mean values ± SEM of two independent experiments (each measured in duplicate) are presented. Significant differences are indicated (*p ≤ 0.05, **p ≤ 0.01, ***p ≤ 0.001)

Although HSF1 knockdown only diminished the MICB expression in H1339 cells, NZ28 strongly reduced the ic and membrane MICA and MICB expression in control as well as in HSF1 knockdown cells (Fig. 4b, c). Therefore, we asked the question whether other transcription factors that have been described to regulate the transcription of MICA/B might also be affected by NZ28. As shown by the luciferase assay, NZ28 inhibited not only the transcriptional activity of HSF1 but also that of NF-κB and Sp1 in H1339 and T47D tumor cells (Fig. 5).

## Discussion

Targeting the heat shock response (Hsp70 or its main transcription factor HSF1) by small molecules or RNA interference has been shown to sensitize tumor cells to Hsp90 inhibition [1–5]. Previous work of our group revealed that HSF1 inhibition by NZ28 increases the radiosensitizing efficiency of the Hsp90 inhibitor NVP-AUY922 in lung and breast cancer cell lines (unpublished data). In recent years, evidence has accumulated that not only the interplay between radio- and chemotherapy, but also the host immune system plays an important role in determining the clinical outcome of cancer patients [23]. Radiochemotherapy has been reported to affect the antigen expression on tumor cells and the secretion of soluble factors that contribute to immune stimulation [24]. Lung and breast tumors are frequently treated with radiotherapy, and NK cells have been documented to be critically involved in anti-tumor immunity against these very common tumor entities [25, 26]. Therefore, in this study, we investigated the effects of this novel combined treatment modality (NZ28 and NVP-AUY922) with respect to NK cell cytotoxicity and NK cell ligand expression on lung and breast tumor cells.

Whereas NVP-AUY922 was shown to increase the activity of HSF1 and the icMICB expression, it did not alter the icMICA expression, suggesting that MICA is not regulated by HSF1 in our investigated cell lines. Indeed, HSF1 knockdown reduced the expression of MICB but not that of MICA. In line with our results, Venkataraman et al. [12] have shown that MICA is less inducible by stress than MICB due to a weaker binding capacity of HSF1 to the HSE.

In contrast to icMICB, the MICB cell surface density was not increased by NVP-AUY922. This might be due to a default in the membrane translocation of MICB. Consistently, the sensitivity of tumor cells toward NK cell-mediated lysis was not improved. Other groups demonstrated that high concentrations of the Hsp90 inhibitor 17-AAG (1 and 6 µM) up-regulate the cell surface density of MICA and MICB on multiple myeloma cells and Hodgkin’s lymphoma cells and thereby can enhance NK cell cytotoxicity [6, 7]. The discrepancy to their results might be either explained by differences in the tumor cell lines or by the lower and more physiological concentration of the Hsp90 inhibitor used in our study.

NZ28 down-regulated the HSF1 activity and the MICA/B expression on lung and mammary tumor cells, and this correlated with a reduced sensitivity to NK cell-mediated lysis. As NZ28 treatment also reduced the MICA and MICB expression in HSF1-depleted cells, we assume that NZ28 exerts its effects on MICA/B expression not only via HSF1. Luciferase reporter assays revealed that the activity of other transcription factors such as Sp1 and NF-κB was also inhibited by NZ28. In line with our results, treatment of Hela cells with a NF-κB inhibitor also reduced the MICA expression [27]. In contrast, in Jurkat cells NF-κB down-regulation by siRNA did not modify the MICA/B expression but silencing of Sp1 reduced the MICA/B expression [14].

Like NZ28, the naturally derived compound triptolide has originally been shown to inhibit the heat shock response [28]. Later on, Banerjee et al. [29] demonstrated that triptolide reduces the activity of Sp1 and this in turn leads to decreased HSF1 and NF-κB activities. By silencing Sp1, they could clearly show that Sp1 is a master regulator for HSF1 and NF-κB. Therefore, we propose that NZ28 might inhibit the transcriptional activity of Sp1 which in turn results in a reduced HSF1 and NF-κB activity. Subsequently, this leads to the down-regulation of both MICA and MICB which causes a complete loss in NK cell-mediated killing.

**Fig. 6:**
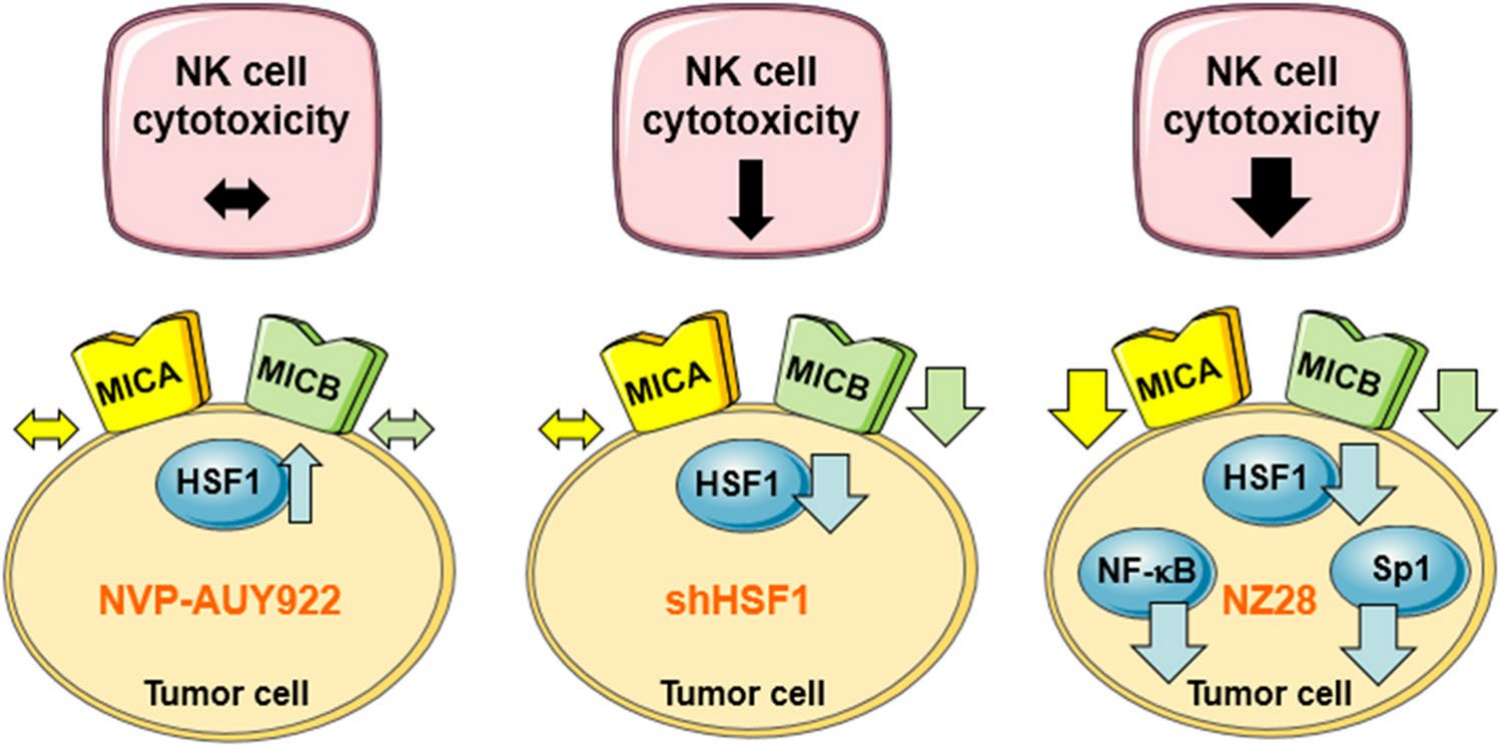
Model depicting the effects of NVP-AUY922, HSF1 knockdown (shHSF1) and NZ28 on HSF1 activity and MICA/B surface density in tumor cells and the associated NK cell cytotoxicity.  indicates no change,  increase,  decrease,  strong decrease

In summary, our data reveal that HSF1 activation by NVP-AUY922 neither affects MICA/B surface density nor affects the associated NK cell cytotoxicity (Fig. 6). However, HSF1 depletion decreases the MICB but not MICA membrane expression and thereby leads to a reduction in the NK cell-mediated cytotoxicity. In contrast, NZ28 severely blocks NK cell-mediated cytotoxicity by a substantial down-regulation of both NK2D ligands, MICA and MICB. This effect might be explained by the fact that NZ28 impairs not only HSF1 but also Sp1 and NF-κB activities.

Despite these negative effects of NZ28 on the function of NK cells, NZ28 has been shown to be very efficient in sensitizing tumor cells toward radiation (unpublished data) and chemotherapy [5].

## Electronic supplementary material

Below is the link to the electronic supplementary material.

Supplementary material 1 (PDF 286 kb)

## Abbreviations

BMBF: Bundesministerium für Bildung und Forschung
DFG: Deutsche Forschungsgemeinschaft
DSMZ: Deutsche Sammlung von Mikroorganismen und Zellkulturen
FACS: Fluorescence-activated cell sorting
HSF1: Heat shock factor 1
Hsp: Heat shock protein
ic: Intracellular
IL-2: Interleukin 2
MICA/B: Major histocompatibility class I chain-related proteins A and B
MFI: Mean fluorescence intensity
NKG2D: Natural killer group 2D
NF-κB: Nuclear factor kappa-light-chain-enhancer of activated B cells
NK: Natural killer
PI: Propidium iodide
Sp1: Specificity protein 1
