# Supplementary material for: NZ28-induced inhibition of HSF1, SP1 and NF-κB triggers the loss of the natural killer cell-activating ligands MICA/B on human tumor cells
Source: Cancer Immunol Immunother. 2015 Feb 18;64(5):599–608. doi: 10.1007/s00262-015-1665-9 (PMC4412431; doi:10.1007/s00262-015-1665-9)
Supplement: Supplementary file 1 — Supplementary material 1 (PDF 286 kb) [file 262_2015_1665_MOESM1_ESM.pdf]

Supplementary Figure 1

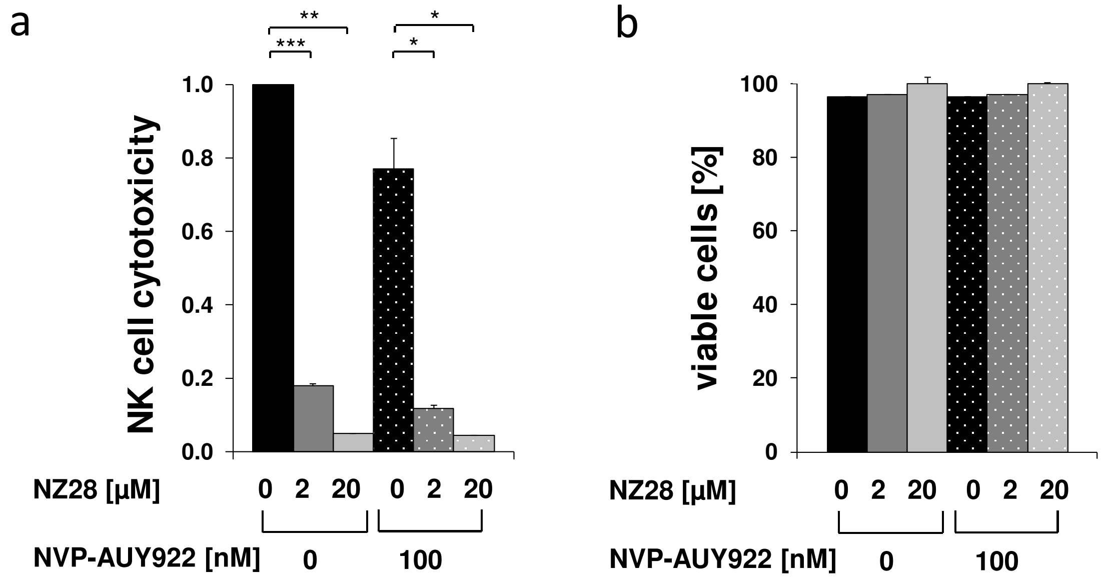

Supplementary Figure 1

T47D tumor cells were treated with NZ28 and/or NVP-AUY922 for 24 h. NK cells were stimulated for 4 days with 100 U/ml IL-2.

a. The cytotoxicity of activated NK cells was measured after a 4 h co-incubation with T47D tumor cells by the CD107a degranulation assay. The relative cytotoxicity of activated NK cells incubated with differentially treated tumor cells compared to untreated tumor cells is shown. White dots in the bar graphs indicate treatment with NVP-AUY922. Graphs represent mean values ± SEM. $^*p \leq 0.05$, $^{**}p \leq 0.01$, $^{***}p \leq 0.001$.

b. T47D tumor cells were stained with propidium iodide (PI) for 1 min. Viable cells (PI negative) were analyzed on a FACSCalibur flow cytometer. White dots in the bar graphs indicate treatment with NVP-AUY922. Mean values ± SEM are shown.

Supplementary Figure 2

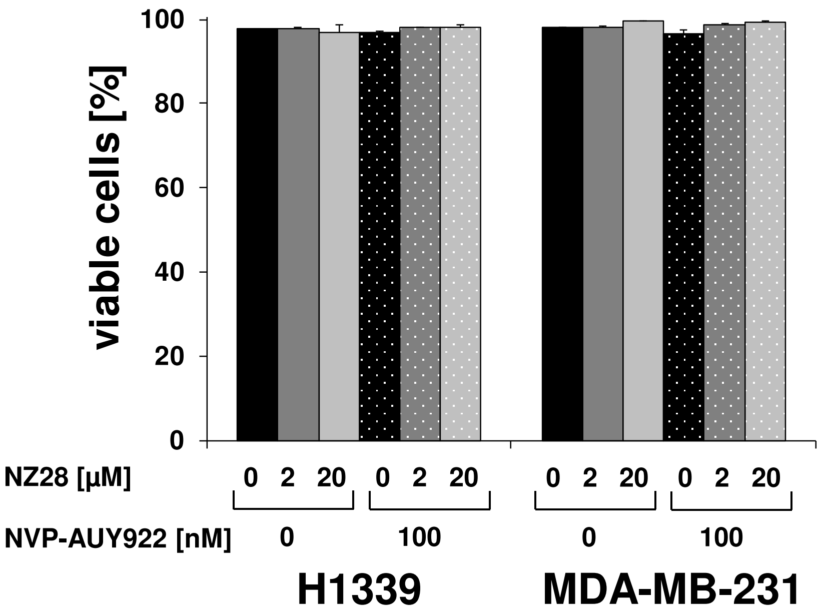

Supplementary Figure 2
H1339 and MDA-MB-231 tumor cells were treated with NZ28 and/or NVP-AUY922 for 24 h and stained with propidium iodide (PI) for 1 min. Viable cells (PI negative) were analyzed on a FACSCalibur flow cytometer. White dots in the bar graphs indicate treatment with NVP-AUY922. Mean values ± SEM of 2-3 independent experiments are shown.

Supplementary Figure 3

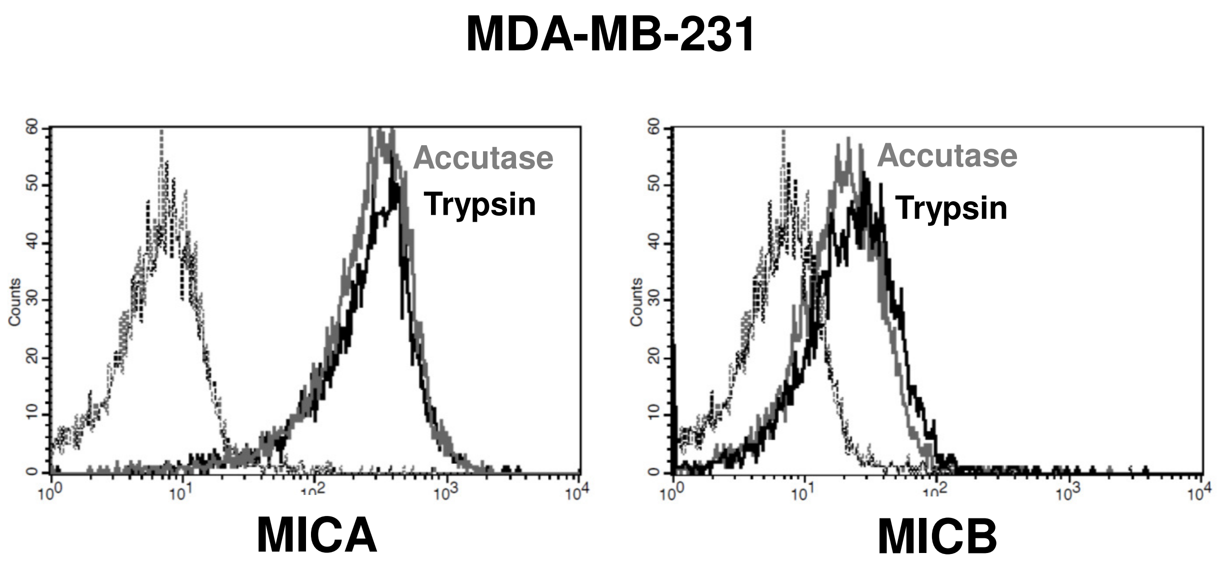

Supplementary Figure 3
MDA-MB-231 tumor cells were stained with antibodies against MICA/B or the respective isotype control. Representative FACS analysis shows MICA (left) and MICB (right) surface staining on tumor cells. The black solid lines represent MICA or MICB staining of cells detached with trypsin and the grey solid lines represent MICA or MICB staining of cells detached with accutase. Dotted lines represent the respective isotype controls.

Supplementary Figure 4

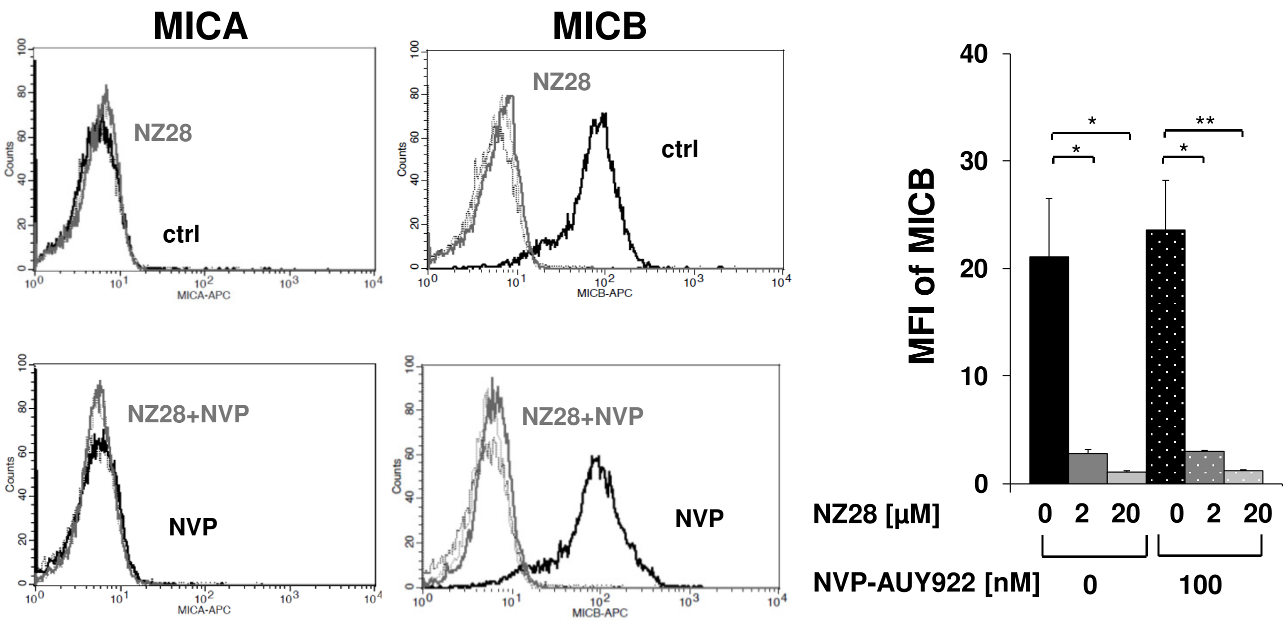

Supplementary Figure 4

T47D tumor cells were treated with NZ28 and/or NVP-AUY922 for 24 h. Tumor cells were stained with antibodies against MICA/B or the respective isotype control. Representative FACS analysis shows MICA (left) and MICB (right) surface staining on tumor cells. The black solid lines represent MICA or MICB staining of untreated cells (ctrl; upper panels) or cells treated with 100 nM NVP-AUY922 (NVP; lower panels). The grey solid lines represent MICA or MICB staining of cells treated with 20 µM NZ28 (NZ28; upper panels) or with 20 µM NZ28 plus 100 nM NVP-AUY922 (NZ28+NVP; lower panels). Dotted lines represent the respective isotype controls. Graphs show mean values ± SEM of the MICB mean fluorescence intensity (MFI). White dots in the bar graphs indicate treatment with NVP-AUY922. *$p \leq 0.05$, **$p \leq 0.01$, ***$p \leq 0.001$.

Supplementary Figure 5

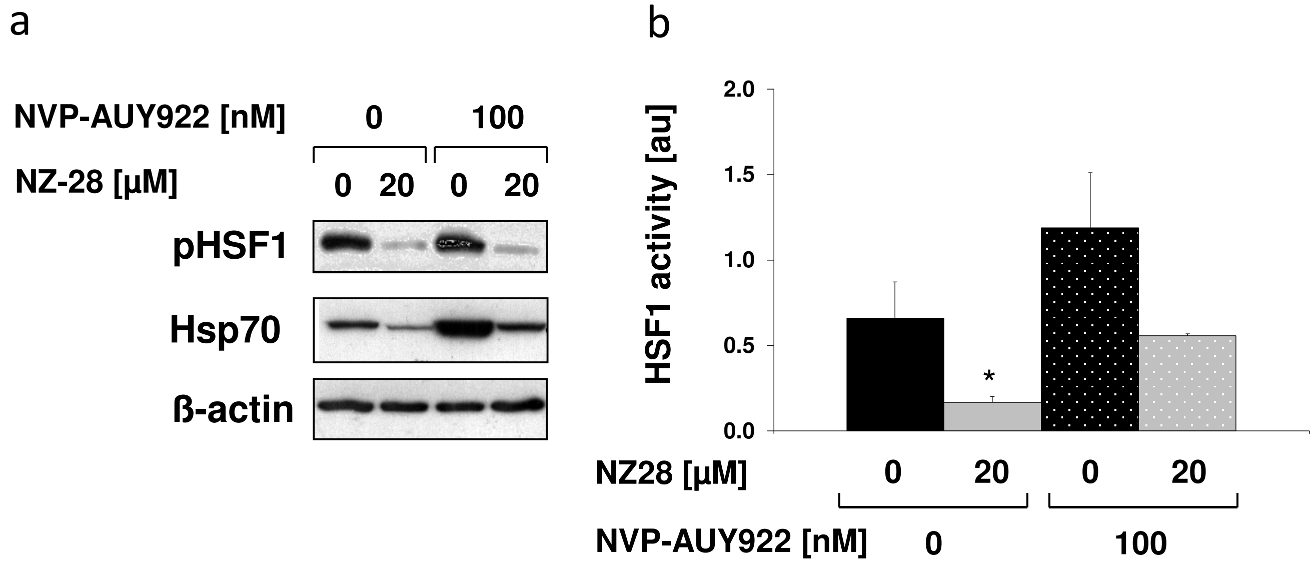

Supplementary Figure 5
a. Representative pHSF1 (phosphoHSF1) and Hsp70 immunoblots of T47D tumor cells treated with 20 µM NZ28 and/or 100 nM NVP-AUY922 for 24 h.
b. Luciferase assay of T47D cells transfected with a HSF1 responsive firefly luciferase construct and treated with 20 µM NZ28 and/or 100 nM NVP-AUY922 for 24 h. Graphs represent mean values ± SEM of at least 3 independent experiments. White dots in the bar graphs indicate treatment with NVP-AUY922. Significant differences are indicated (*$p \leq 0.05$).
